# Repurposing drugs to advance the treatment of Buruli ulcer

**DOI:** 10.1128/aac.00029-25

**Published:** 2025-03-26

**Authors:** Sanjay Singh, Rie Yotsu, Eric Nuremberger, Shashikant Srivastava

**Affiliations:** 1 Division of Infectious Diseases, Department of Medicine, School of Medicine, University of Texas Health Science Centre at Tyler 12341, Tyler, Texas, USA; 2 Department of Tropical Medicine and Infectious Disease, School of Public Health and Tropical Medicine, Tulane University 685162, New Orleans, Louisiana, USA; 3 Center for Tuberculosis Research, Division of Infectious Diseases, Johns Hopkins University School of Medicine 229385, Baltimore, Maryland, USA; 4 Department of Cellular and Molecular Biology, Center for Biomedical Research, University of Texas Health Science Centre at Tyler 12341, Tyler, Texas, USA; St. George's, University of London, London, United Kingdom

**Keywords:** *Mycobacterium ulcerans*, autoluminescence, Buruli ulcer, drug susceptibility, repurposing

## Abstract

Aligned with the World Health Organization’s Road Map, there is an unmet need for research to improve the treatment of Buruli ulcer caused by *Mycobacterium ulcerans*. The repurposing of drugs could speed up new regimen development to treat Buruli ulcer. Using a virulent reporter strain of *M. ulcerans* with intrinsic bioluminescence (MuAL), we compared the minimum inhibitory concentration (MIC) of moxifloxacin, bedaquiline, telacebec, tebipenem, omadacycline, and epetraborole with standard-of-care drugs—rifampin and clarithromycin. We also compared the efficacy (maximal kill or *E*_max_) and potency (EC_50_ or concentration associated with 50% of *E*_max_) as single and two-drug combinations. The doubling time of MuAL was calculated as 3.66 (95% CI: 3.41–3.93) days. Telacebec had the lowest MIC (0.0000075 mg/L) among the eight drugs tested, followed by rifampicin (0.5 mg/L) and clarithromycin (0.5 mg/L). Epetraborole, telacebec, and moxifloxacin monotherapy at tested concentrations showed higher *E*_max_ compared to clarithromycin and rifampicin. In preclinical studies, telacebec combined with rifampicin or epetraborole and epetraborole combinations with moxifloxacin and omadacycline were superior to the rifampin-clarithromycin combination. The MuAL strain is useful in the rapid screening of drugs’ efficacy and potency against *M. ulcerans*. We should leverage the progress made in the tuberculosis drug development pipeline to repurpose the drugs for the rapid development of new therapeutic modalities for Buruli ulcer.

## INTRODUCTION

Buruli ulcer is a chronic disease caused by a bacterium, *Mycobacterium ulcerans* (*Mul*)*,* which belongs to the family of mycobacteria, including the organisms that cause tuberculosis and leprosy. Globally, Buruli ulcer cases have been reported in at least 33 countries, and at least 14 countries regularly report data to the World Health Organization (WHO) (1–3). The disease is endemic in the African Region, namely countries like Côte d'Ivoire, Benin, Ghana, the Democratic Republic of the Congo, Cameroon, Nigeria, Togo, and Liberia (2). With increasing globalization, cases are not strictly confined to endemic areas, and imports of cases have been documented in non-endemic regions (4). Most cases occur in children aged 5–15 years, except in Australia and Japan, where the disease is prevalent in adults >50 years (1, 5). *Mul* produces mycolactone, a macrolide toxin that is the primary cause of skin necrosis and other symptoms characteristic of the disease (1, 6). Buruli ulcer can lead to long-term deformities and disabilities. Therefore, early detection of cases and treatment is crucial. Once large ulcers develop, antibiotic treatment alone cannot cure the disease, and additional interventions, including wound management and surgery (e.g., debridement and skin grafting), are required to speed up the healing process.

The recommended treatment for Buruli ulcer consists of a combination of rifampicin (10 mg/kg of body weight once daily) and clarithromycin (7.5 mg/kg of body weight twice daily) for at least 8 weeks (7), which is widely used in most endemic countries and provided through WHO (7). In some countries, like Australia, a combination of rifampin (10 mg/kg of body weight once daily) and moxifloxacin (400 mg once daily) or ciprofloxacin (500 mg twice daily) is also used (8). However, the effectiveness of this regimen has not been tested in randomized trials (7, 9). A new combination of rifampin-clarithromycin-amoxicillin/clavulanate administered for 4 weeks has also been advanced to a clinical trial comparing it to the 8-week rifampin-clarithromycin combination to shorten the therapy duration (7, 10). True to the WHO road map and research priorities (11), there is an unmet need for research studies to improve the treatment of Buruli ulcer. Given the very high cost of developing a drug and the lengthy approval process (12), repurposing drugs that are developed to treat other bacterial infections could expedite new therapeutics development for Buruli ulcer.

Significant impediments to the prevention and treatment of Buruli ulcer include the lack of information on its transmission mode and replicative reservoirs. *Mul* grows exceptionally slowly in the laboratory, and it requires up to 3 months or more to form countable colonies on solid media. This slow growth rate is one of the biggest hurdles in discovering and developing new drugs to improve the treatment of Buruli ulcer. Here, we performed experiments on minimum inhibitory concentration (MIC), concentration-response, and two-drug combination efficacy with moxifloxacin, tebipenem, bedaquiline, telacebec, omadacycline, and epetraborole, comparing the efficacy of these drug candidates alone or in combination with rifampin and clarithromycin, using a reporter strain of *Mul* with intrinsic bioluminescence (13–15). The study drugs were selected based on oral bioavailability and their status as a drug currently recommended (e.g., moxifloxacin, bedaquiline, omadacycline) and/or studied in Phase II or III clinical trials (e.g., telacebec and epetraborole) for treatment of mycobacterial infections. In the case of tebipenem, it is a drug in Phase III clinical trial for other infectious indications that belongs to a drug class with known anti-mycobacterial activity. As such, there is a wealth of population pharmacokinetics, as well as safety and toxicity data, available for these drugs to make an informed decision to advance these for the treatment of Buruli ulcer.

## MATERIALS AND METHODS

We used a virulent reporter strain of *Mul* with intrinsic bioluminescence (13–15) in all experiments. As previously described, the *Mul* strain was originally isolated from a patient in Ghana and was subsequently genetically engineered for intrinsic bioluminescence. Drug susceptibility assays using relative light units (RLUs) as a surrogate measure for bacterial viability produced results that correlated closely with colony-forming unit (CFU) counts for a panel of drugs with various mechanisms of action (13–15). Middlebrook 7H9 broth (herein termed “broth”) and Middlebrook 7H10 agar (herein termed “agar”), both supplemented with 10% oleic acid, albumin, dextrose, and catalase, were used as culture media. Kanamycin (50 mg/L) was also added to the media to maintain the selection of the autoluminescent *Mul* strain (MuAL). The luminescence was measured using a benchtop luminometer (GLOMAX 20/20 luminometer, Promega, USA). Drugs (rifampin, clarithromycin, moxifloxacin, tebipenem, bedaquiline, telacebec, omadacycline, and epetraborole) were purchased from BOC Sciences (Sheryl, NY, USA).

First, we performed experiments to determine the growth rate of MuAL. To prepare the inoculum, RLU of the logarithmic phase growth culture was adjusted to 20,000 RLU, and a total of 1 mL was dispensed in sterile 1.8 mL tubes. Cultures were incubated at 32°C, and RLU was measured every 3 days for 24 days. The experiment was performed in triplicate, and each tube was read three times; thus, nine RLU readouts per time point. In addition, 200 µL of the inoculum was spread on agar plates and incubated at 32°C for up to 100 days to record the appearance of visible colonies. The agar plates were sealed in a Ziplock bag to prevent the drying of agar over prolonged periods of incubation.

Second, in the MIC experiments, the drug and concentration ranges, in two-fold dilution, were as follows: rifampin, 0.0075–1 mg/L; clarithromycin, 0.015–4 mg/L; moxifloxacin, 0.06–8 mg/L; tebipenem, 0.06–4 mg/L; bedaquiline, 0.005–0.125 mg/L; telacebec, 0.0000004–0.00012 mg/L; omadacycline, 0.015–8 mg/L; and epetraborole, 0.03–4 mg/L. Inoculum was prepared as described above, and the drug and bacteria were co-incubated at 32°C in 1.8 mL tubes. RLU was measured every 3 days for up to 24 days. The experiment was performed in duplicate, and each tube was read three times; thus, six RLU readouts per time point. The lowest drug concentration showing a 2 log_10_ decrease in RLU compared to the nontreated controls was recorded as the MIC.

Third, we performed static concentration-response studies with each drug using the concentration ranges and inoculum preparation mentioned above. Based on the MIC experiments, day 9 was selected to record the RLU data. There were two replicates per drug concentration, and RLU for each tube was measured three times. The inhibitory sigmoid maximal effect model was used to describe the relationship between the drug concentrations and the RLU as a surrogate for the bacterial burden.

Fourth, we tested the efficacy of each drug alone or in combination at concentrations achieved with a standard human dose (Table 1). Bacterial kill (i.e., decrease in RLU compared to day 9 nontreated controls) was used to rank the drugs as monotherapy and two-drug combinations as backbone regimens for future multidrug regimen development.

**TABLE 1:** MIC of the study drugs and concentrations used in the experiments

Drug	Concentration used in combination studies (mg/L)	MIC (mg/L)
Rifampin	2	0.5
Clarithromycin	0.58	0.5
Moxifloxacin	4.2	2
Bedaquiline	1.2	0.06
Telacebec	0.000131	0.0000075
Omadacycline	0.81	0.25
Epetraborole	3	0.125
Tebipenem	24	2

Data for all experiments from the luminometer was collected using GLOMAX SIS version 1.10.0 software (Promega, USA), exported to MS-EXCEL for log_10_ transformation, and subsequently used for the exponential growth model, inhibitory sigmoid model fit, linear regression, and graphing, where applicable, using GraphPad Prism (version 10).

## RESULTS

**Fig 1:**
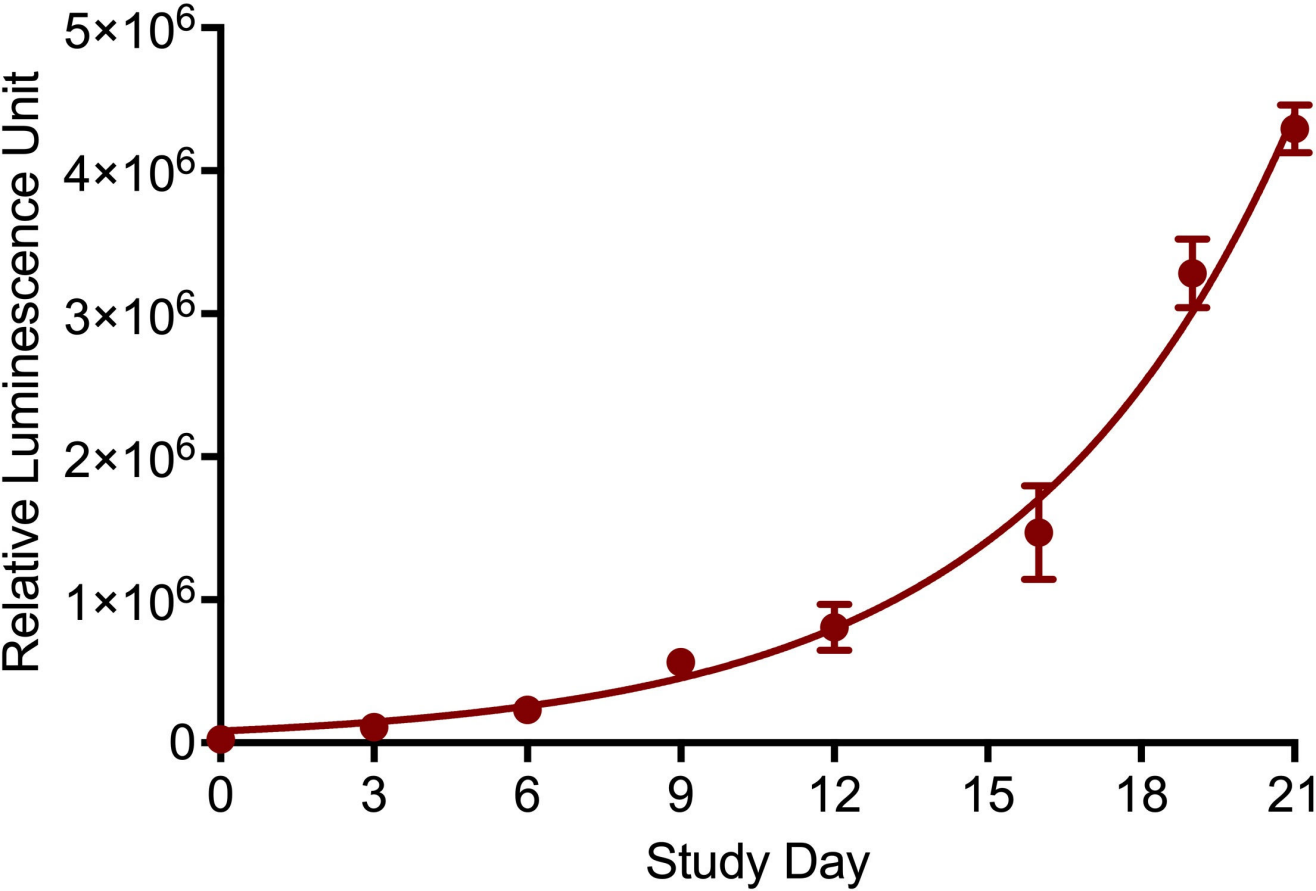
Growth characteristics of autoluminescent *M. ulcerans* (MuAL). Nine RLU readouts per time point were used to calculate the MuAL strain doubling time as 3.66 (95% CI: 3.41–3.93) days.

Figure 1 shows the change in the RLU of MuAL cultures over 21 days. An increase in the RLU indicates the growth of the bacteria. The doubling time of MuAL was calculated as 3.66 (95% CI: 3.41–3.93) days. The MIC of each drug against the MuAL strain, based on the 2 log_10_ decrease in RLU compared to the nontreated controls, recorded after 9 days of incubation, is listed in Table 1.

**Fig 2:**
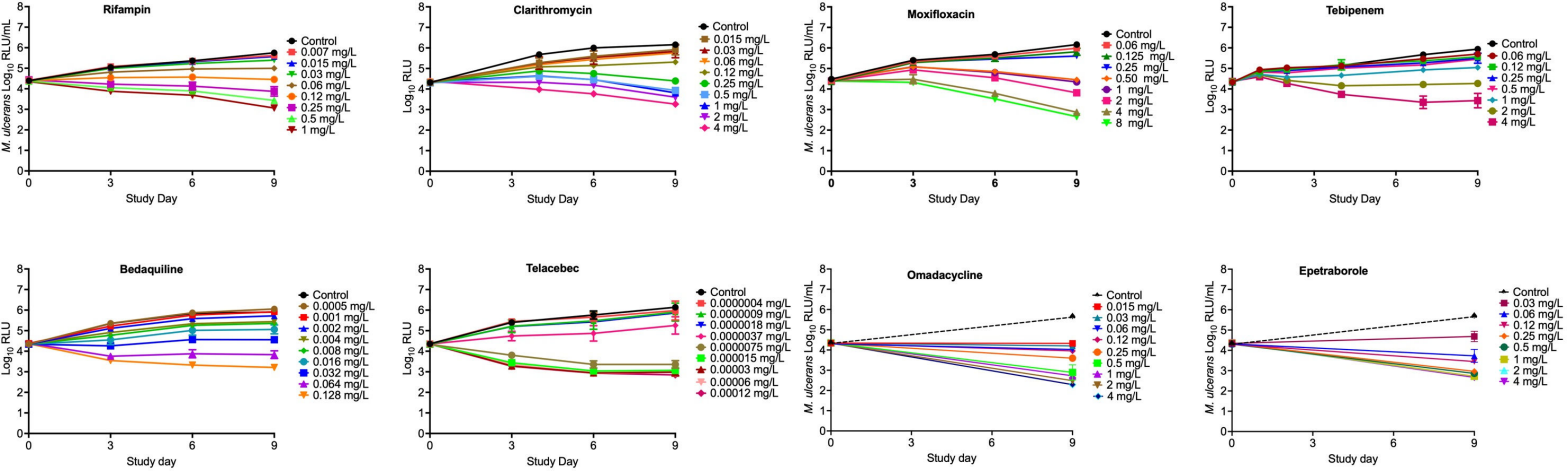
Static time-kill curves of drugs against *M. ulcerans*. Relative light units were measured for up to 21 days. However, for the clarity of graphs, the *x*-axis is truncated to day 9, the time point after which kill trajectories did not change.

**Fig 3:**
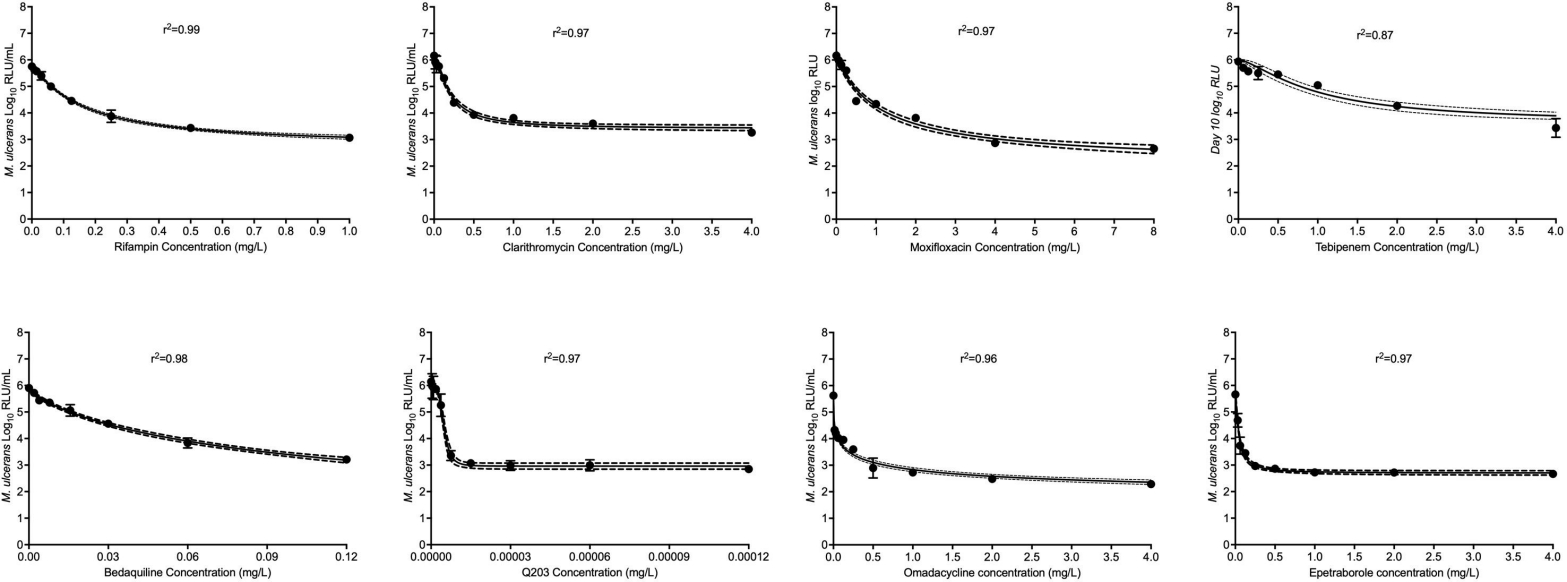
Inhibitory sigmoid model fits showing the relationships between drug concentrations and *M. ulcerans* relative light units. Solid line is model fit, and dotted lines represent 95% confidence intervals.

Figure 2 shows the kill curve of the study drugs with each concentration after 9 days of incubation. As shown in the figure, several concentrations of each drug were able to keep the RLU below stasis. Results of the concentration-response studies, analyzed using the four-parameter inhibitory sigmoid maximal effect model, are shown in Fig. 3; Table 2. Based on the potency (EC_50_ or concentration associated with 50% of the maximal effect) of the drugs against MuAL, the drugs were ranked in the following order: telacebec (EC_50_ = 0.000005 mg/L) > epetraborole (EC_50_ = 0.05 mg/L) > bedaquiline (EC_50_ = 0.11 mg/L) > rifampin (EC_50_ = 0.16 mg/L) > omadacycline (EC_50_ = 0.20 mg/L) > clarithromycin (EC_50_ = 0.20 mg/L) > tebipenem (EC_50_ = 0.97 mg/L) > moxifloxacin (EC_50_ = 1.12 mg/L).

**TABLE 2:** Inhibitory sigmoid maximal effect model parameters describing drug efficacy and potency^*a*^^,^^*b*^

Drug	*E*_con_ (log_10_ RLU)	*E*_max_ (log_10_ RLU)	*H*	EC_50_ (mg/L)	*r* ^2^
Rifampin	5.75 (5.68–5.82)	3.00 (2.76–3.25)	1.13 (0.98–1.29)	0.16 (0.14–0.19)	0.99
Clarithromycin	6.07 (5.96–6.18)	2.66 (2.47–2.85)	1.46 (1.18–1.75)	0.20 (0.17–0.23)	0.97
Moxifloxacin	6.23 (6.06–6.39)	4.18 (3.57–4.79)	0.92 (0.72–1.13)	1.12 (0.74–1.50)	0.97
Tebipenem	5.94 (5.73–6.00)	2.50 (2.23–2.78)	1.43 (1.09–1.76)	0.97 (0.80–1.14)	0.87
Bedaquiline	5.88 (5.78–5.98)	5.14 (2.89–7.39)	0.82 (0.62–1.02)	0.11 (−0.0007–0.2133)	0.98
Telacebec	5.99 (5.85–6.10)	3.03 (2.86–3.19)	4.15 (3.06–5.24)	0.000005 (4.39e-006–5.27e-006)	0.97
Omadacycline	5.60 (5.43–5.76)	4.21 (3.50–4.91)	0.40 (0.30–0.49)	0.20 (0.02–0.38)	0.96
Epetraborole	5.68 (5.53–5.82)	2.98 (2.81–3.15)	1.32 (1.08–1.56)	0.05 (0.04–0.06)	0.97

^*a*^ *E*_con_ is the relative light units in nontreated controls. *E*_max_ is the difference in RLU between the nontreated controls and maximum drug concentration (i.e., efficacy). The potency of the drug (EC_50_) is the drug concentration required to exert 50% of the *E*_max_. *H* is the Hill’s coefficient, which is dimensionless.

^*b*^ Data are presented as mean and 95% confidence intervals. Experiments were performed in duplicate per concentration, and RLU was measured three times per tube. Thus, there were two biological and six technical replicates per concentration.

**Fig 4:**
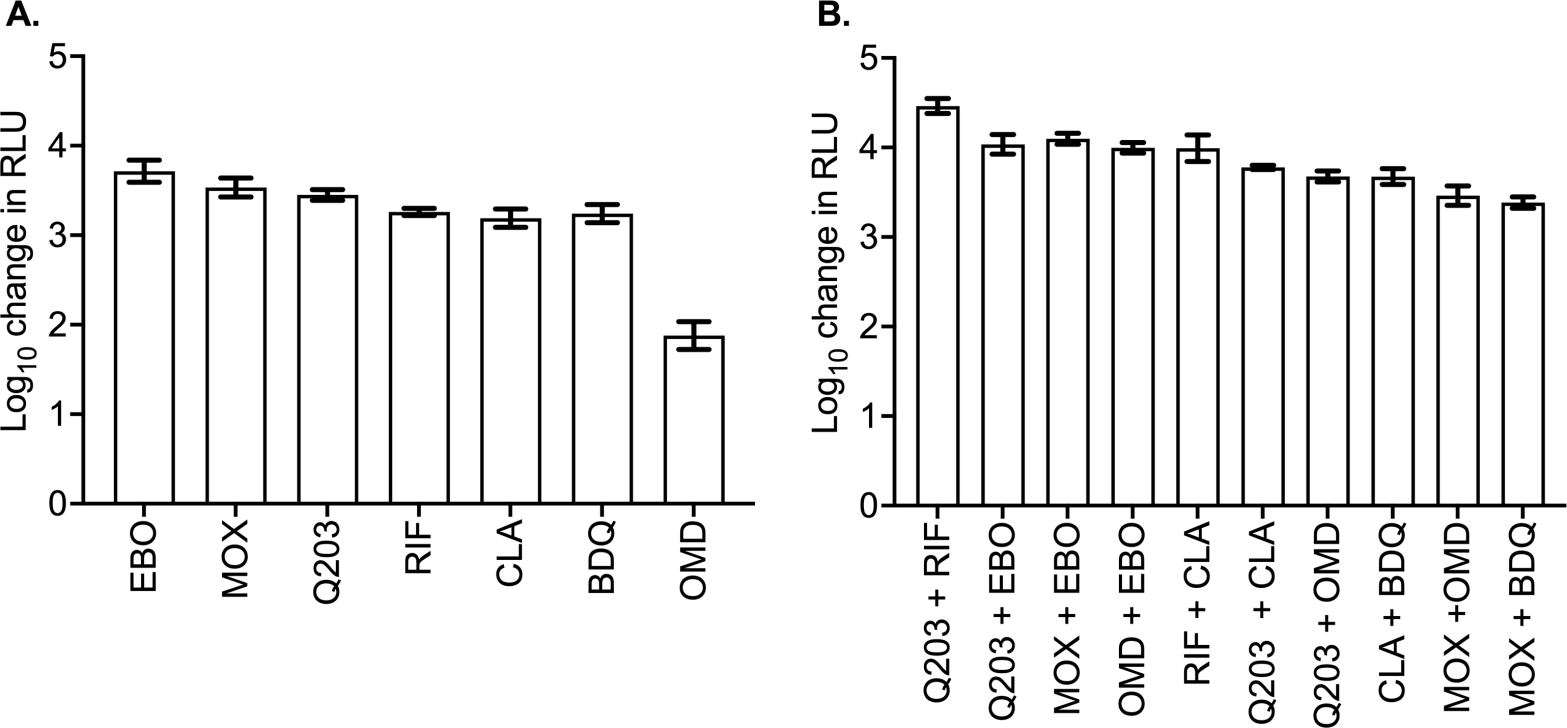
The rank order of drugs (**A**) alone and (**B**) as two-drug combinations for *M. ulcerans* kill at tested concentrations. The *y*-axis represents the difference in relative light units (transformed to log_10_) compared to the nontreated control with each drug on study day 9. CLA, clarithromycin; BDQ, bedaquiline; RIF, rifampin; Q203, telacebec; OMD, omadacycline; EBO, epetraborole; and MOXI, moxifloxacin.

The results of the monotherapy and two-drug combination studies, with peak concentration achieved with standard doses, are shown in Fig. 4A and B; Tables 3 and 4, where analysis of variance was used to compare the different drugs and combinations. As shown in Fig. 4A, epetraborole, telacebec, and moxifloxacin outranked the standard-of-care drugs (rifampin and clarithromycin). Table 3 shows the relative magnitude of the bacterial kill (measured as the decline in log_10_ RLU) using rifampin as the comparator. There was no significant difference between rifampin and either clarithromycin or bedaquiline. However, epetraborole, moxifloxacin, and telacebec showed significantly better killing compared to rifampin, while omadacycline showed significantly worse killing. Figure 4B; Table 4 compare the experimental combinations in terms of bacterial kill measured as a decline in log_10_ RLU relative to that observed with rifampin-clarithromycin. At the tested concentrations, except for telacebec-epetraborole, moxifloxacin-epetraborole, and omadacycline-epetraborole, all two-drug combinations were significantly different from the standard rifampin-clarithromycin combination.

**TABLE 3:** Relative activity of drugs proposed for repurposing^*a*^^,^^*b*^

Drugs	Mean difference	95% CI of difference	Summary	Adjusted *P* value
Clarithromycin vs rifampin	−0.07000	−0.2386 to 0.09860	ns	>0.9999
Telacebec vs rifampin	0.1900	0.02140 to 0.3586	*	0.0199
Omadacycline vs rifampin	−1.382	−1.550 to −1.213	****	<0.0001
Epetraborole vs rifampin	0.4550	0.2864 to 0.6236	****	<0.0001
Moxifloxacin vs rifampin	0.2733	0.1047 to 0.4419	***	0.0004
Bedaquiline vs rifampin	−0.01833	−0.1869 to 0.1503	ns	>0.9999

^*a*^ Rifampin was used as a comparator.

^*b*^ CI, 95% confidence intervals.

**TABLE 4:** Relative activity of experimental two-drug combinations compared to the rifampin-clarithromycin combination^*a*^

Drug combinations	Mean difference	95% CI of difference	Summary	Adjusted *P* value
CLA + BDQ vs RIF + CLA	−0.3167	−0.4612 to −0.1721	****	<0.0001
Q203 + RIF vs RIF + CLA	0.4733	0.3288 to 0.6179	****	<0.0001
Q203 + CLA vs RIF + CLA	−0.2117	−0.3562 to −0.06713	***	0.0009
Q203 + OMD vs RIF + CLA	−0.3150	−0.4595 to −0.1705	****	<0.0001
Q203 + EBO vs RIF + CLA	0.04333	−0.1012 to 0.1879	ns	>0.9999
MOXI + EBO vs RIF + CLA	0.1067	−0.03787 to 0.2512	ns	0.3361
MOXI + OMD vs RIF + CLA	−0.5317	−0.6762 to −0.3871	****	<0.0001
OMD + EBO vs RIF + CLA	0.005000	−0.1395 to 0.1495	ns	>0.9999
MOXI + BDQ vs RIF + CLA	−0.6067	−0.7512 to −0.4621	****	<0.0001

^*a*^ CLA, clarithromycin; BDQ, bedaquiline; RIF, rifampin; Q203, telacebec; OMD, omadacycline; EBO, epetraborole; MOXI, moxifloxacin; and CI, 95% confidence intervals.

## DISCUSSION

*M. ulcerans*, the etiological agent of Buruli ulcer, remains a neglected pathogen, with limited investment in research and drug development. There is an urgent need to develop new drugs and combinations with pharmacokinetics/pharmacodynamics-optimized doses to reduce the therapy duration and the healing time for ulcers. However, as there are a limited number of patients (2,000–5,000 annual cases globally), the conduct of clinical trials to test new treatments for Buruli ulcer is challenging. The very high cost to develop a new drug is another disincentive to pharmaceutical companies to focus on neglected tropical diseases, including Buruli ulcer (12). Therefore, repurposing drugs developed to treat other bacterial infections, including tuberculosis (16), could be the best strategy to expedite the identification and development of new therapeutic candidates for the treatment of Buruli ulcer. The drugs tested in the present study are either approved for clinical use or studied in late-stage clinical trials as anti-infectives and have been shown to be active against mycobacteria (17–31), including multi-drug-resistant *Mycobacterium tuberculosis*, *Mycobacterium avium* complex, *Mycobacterium abscessus*, and *Mycobacterium kansasii*, and the results presented here highlight the therapeutic potential against *M. ulcerans*.

One of the biggest hurdles in discovering and developing new drugs for the treatment of Buruli ulcer is the slow growth and very long incubation time (up to 3 months to form countable colonies on solid media) for *M. ulcerans*. Here, we report the use of a reporter strain of *M. ulcerans* with intrinsic bioluminescence that could fast-track pre-clinical studies against *M. ulcerans* (13–15, 32). The measurement of RLUs and their use as a quantitative readout serve as an innovative surrogate marker for viable bacteria (13–15, 32).

First, we showed that the doubling time of the virulent MuAL strain is 3.66 days (95% CI: 3.41–3.93) compared to the *in vivo* doubling time of 4.93 days (95% confidence interval: 3.5–8.3) for wild-type *M. ulcerans* (33). Second, based on the doubling time, we measured the RLUs every 3 days for up to 21 days in the MIC experiments and found that day 9 readouts could be used for reproducible results. Therefore, in subsequent experiments with omadacycline, epetraborole, and in combination studies, day 9 was used as the end point, as opposed to a previous study that used day 7 RLU reading for MIC determination (15). Elsewhere, MICs using MuAL strains were found in agreement with those reported using the standard susceptibility testing in 7H11 agar (13, 15). Thus, our results add to the existing evidence base of MuAL strain being a valuable, reliable, and inexpensive tool to rapidly test the drugs for activity against *M. ulcerans* (13–15, 32).

Third, we used the data to determine the relationship between the drug concentrations and the bacterial burden (RLU measurements). We found that telacebec, moxifloxacin, and epetraborole have better efficacy and potency against *M. ulcerans* compared to the standard-of-care drugs. The existing knowledge on the population pharmacokinetics/pharmacodynamics and long-term safety of these drugs (34–42) could be an advantage for designing future studies to determine the optimal exposure and clinical dose finding in *in vitro* and *in silico* studies, following the standards published elsewhere (43).

Fourth, to determine the therapeutic potential of novel drug combinations, we tested the drugs as two-drug combinations with concentrations that could be acheived with human doses of each drug. Epetraborole, moxifloxacin, and telacebec showed the highest kill at the concentrations tested. Combinations of epetraborole with telacebec, moxifloxacin, or omadacycline showed similar killing effects compared to the rifampin-clarithromycin standard therapy, while the rifampin-telacebec combination was superior to the rifampin-clarithromycin combination, similar to previously reported activity in a mouse model of Buruli ulcer (29). Epetraborole and omadacycline are currently being evaluated for the treatment of non-tuberculous mycobacterial lung infections (NCT05327803 and NCT04922554), while telacebec is being evaluated for the treatment of Buruli ulcer in the TREAT-BU trial (NCT06481163). The available evidence, to an extent, indicates that the 8-week rifampicin backbone standard regimen used in endemic countries might be working in patients (9, 44). However, all patients receive the same dose, while children are treated with weight-based dosing. Evidence to support this practice is lacking, and optimal therapy duration is unknown. Depending on the severity, longer treatment may be needed beyond 8 weeks. There is a need for alternative treatment regimens. Therefore, further systematic studies are needed with the repurposing drug candidates studied here to develop more effective combinations to treat *Mul* infections.

The potential limitations of our studies include the use of a single *M. ulcerans* strain. However, the reporter strain we used has previously been established as a rapid screening tool in preclinical *in vitro* and *in vivo* studies (13–15, 32, 33). Given the time it takes to culture the bacteria on solid media, the use of the MuAL strain and RLU as a surrogate for CFU is a valuable tool in the arsenal to advance the therapeutic development for Buruli ulcer. An additional potential limitation is that the omadacycline experiments were performed without daily drug supplementation (45, 46). Therefore, the activity of omadacycline could be greater than reported here. Further confirmation of the efficacy of selected combinations in an *in vivo* model is warranted.

In conclusion, drug development for neglected and rare diseases, such as Buruli ulcer, has long been overlooked. The use of the reporter *M. ulcerans* strain is an attractive option for rapid screening of drugs to repurpose them for the treatment of Buruli ulcer. We should leverage the progress made in drug development for tuberculosis and other infections to rapidly develop new therapeutic modalities for Buruli ulcer.

## Data Availability

Upon a reasonable request, the raw data for the results presented in the manuscript are available with the corresponding author, following UTHSCT’s data-sharing policy.
