# Erratum for Singh et al., “Repurposing drugs to advance the treatment of Buruli ulcer”

**DOI:** 10.1128/aac.00724-25

**Published:** 2025-06-18

**Authors:** Sanjay Singh, Rie Yotsu, Eric Nuermberger, Shashikant Srivastava

## ERRATUM

Volume 69, no. 5, e00029-25, 2025, https://doi.org/10.1128/aac.00029-25. Page 1, byline: Eric Nuermberger’s surname should appear as shown in this erratum.

Page 8, Author ORCIDs: “Nuremberger” should read “Nuermberger.”

Page 8, Author Contributions: “Nuremberger” should appear as “Nuermberger.”

